# Bibliometric analysis of the common vampire bat (*Desmodus rotundus*) prey in the American continent

**DOI:** 10.22201/fmvz.24486760e.2025.1396

**Published:** 2025-10-21

**Authors:** Juliana Montufar Patiño, Diego Soler-Tovar, Luis E. Escobar

**Affiliations:** 1 Universidad de La Salle., Facultad de Ciencias Agropecuarias., Semillero de Investigación Una Salud., Bogotá, Colombia.; 2 Universidad de La Salle., Facultad de Ciencias Agropecuarias., Grupo Epidemiología y Salud Pública., Bogotá, Colombia.; 3 Virginia Tech. Department of Fish and Wildlife Conservation., Blacksburg, Virginia, United States.

**Keywords:** Geographic distribution, Trophic ecology, Feeding habits, Bat, Sanguivore, Rabies

## Abstract

The common vampire bat (*Desmodus rotundus*) is distributed across the Americas in both natural and agricultural habitats, from northwestern Mexico to northern Argentina. *Desmodus rotundus* feeds exclusively on the blood of various vertebrate species. Its feeding behavior impacts animal and human health due to the risk of rabies virus transmission. The objective of this study was to explore the wild and domestic prey of *D. rotundus* through a systematic review and a bibliometric analysis. Scientific and photographic evidence related to the diet of *D. rotundus* in the Americas was searched and selected from reference databases. Geographic location and biome data were recorded where predation events were documented. The review suggests that *D. rotundus* can inhabit a wide variety of ecosystems, ranging from deserts to tropical forests, and even elevations above 3100 meters. More than 40 wild and domestic species were identified in its diet, ranging from birds such as Humboldt penguins to large predators such as pumas, suggesting that *D. rotundus* is capable of, such as Humboldt penguins, to large predators, including adapting its diet according to prey availability and environmental conditions. The bibliometric analysis highlighted the generalist ecology of *D. rotundus* in terms of feeding behavior, habitat use, and distribution range. This study provides a baseline for identifying general patterns in the diet of *D. rotundus* and quantitatively complements the existing literature on the common vampire bat’s feeding ecology.

## Study contribution

The common vampire bat (*Desmodus rotundus*) plays a significant role in rabies transmission, affecting both animal and human health across a wide geographic range in the Americas. This study provides an analysis of the diet of *D. rotundus*, identifying more than 40 wild and domestic prey species. The findings offer a baseline for understanding the feeding preferences of *D. rotundus* and its dietary flexibility in response to environmental conditions. The research also highlights the importance of including both wild and domestic species in surveillance programs aimed at controlling the spread of zoonotic diseases. Furthermore, it suggests the need to improve monitoring techniques to obtain more accurate data on predator–prey interactions, thereby facilitating the implementation of more effective strategies for rabies prevention and control.

## Introduction

Bats are known to serve as reservoirs for various pathogenic agents, many of which cause zoonotic diseases.^(1)^ They play a very important role in the transmission of the rabies virus.^(2)^ The study and understanding of the ecology of bat-transmitted rabies virus are important due to its impact on animal and public health.^(3)^ Of the 1 200 bat species reported, only three are sanguivorous: *Desmodus rotundus* (Phyllostomidae, E. Geoffroy, 1810), *Diphylla ecaudata* (Phyllostomidae, Spix, 1823) and *Diaemus youngi* (Phyllostomidae, Jentink, 1893). These sanguivorous bat species are found exclusively in the American continent,^(4)^ with *D. rotundus* beings the most common and extensively studied.^(5)^

The feeding habit of *D. rotundus* requires biting its prey, which facilitates the transmission of the rabies virus and other pathogens.^(3)^ The most effective strategies for controlling rabies transmitted by *D. rotundus* in Latin America are based on the preventive vaccination of humans and cattle.^(6)^ However, the reduction of bat populations through widespread, non-selective killing is still widely practiced by livestock producers and authorities.^(5)^ In the absence of comprehensive policies and education on rabies prevention, the World Health Organization has concluded that rabies transmitted by bats is difficult to eradicate.^(7)^

Currently, livestock and human vaccination campaigns are mostly reactive. Although vaccination in humans and cattle is effective as a prophylactic method,^(8)^ vaccination programs are implemented in response to outbreaks.^(9)^ In developing Latin American countries, proactive vaccination remains an economic and logistical challenge.^(8)^ Anthropogenic activities such as urbanization and the expansion of agricultural and livestock frontiers disrupt ecological functions.^(10)^ These ecological disturbances alter biodiversity composition,^(11)^ and changes in species composition within an area can modify the prey availability for *D. rotundus*.^(12)^ Historical records suggest that *D. rotundus* prefers cattle over any wild mammal species.^(13)^

The shift of *D. rotundus* from wild prey to livestock offers new opportunities for pathogen transmission.^(10)^ Predation on cattle may result in secondary cases of rabies in humans and domestic animals.^(14)^ Rabies control programs began to be organized in Latin America and the Caribbean in the 1970s,^(8)^ and since then, surveillance and control measures for rabies virus transmission have been implemented.^(15)^ The “One Health” approach has been suggested as an effective framework to reduce rabies cases for considering virus circulation among both wild and domestic animals, while also taking into consideration the surrounding landscape conditions.^(16)^

The objective of this study was to explore the diversity of wild and domestic prey of *D. rotundus* across its geographic distribution using a bibliographic analysis. The data compiled here may provide a foundation for future research on the prevention and control of sylvatic rabies, serving as a tool to help prioritize areas and species at risk of *D. rotundus* attacks.

## Materials and Methods

### Data Collection

To gather information on the diet of *Desmodus rotundus*, a systematic literature review was conducted using several scientific databases. The databases consulted include Dialnet, PubMed, Redalyc, SciELO, ScienceDirect, Scopus, Springer Books-Journal, and Web of Science. The search terms used were: “*Desmodus rotundus*”, “bat rabies”, “diet”, and “host preference”. For Scopus, the following search strategy was applied: ((“Common Vampire Bat”) OR (“*Desmodus rotundus*”)) AND ((“prey”) OR ((“diet behavior”) OR (“diet”) OR (“bat rabies”))). This search was applied to the title, abstract, and keyword fields and was performed on September 7, 2023, with no restrictions regarding country, year, or language of publication.

Additionally, complementary sources such as ResearchGate (https://www.researchgate.net/) and official institutional websites available online were consulted using the same search terms. Audiovisual materials (videos and photographs) from open-access platforms such as YouTube and camera traps were also considered, provided they met the following inclusion criteria: clear visual evidence of a *D. rotundus* attack, identification of the prey species or habitat, and an estimated or explicit geographic location. No restrictions were applied regarding the country, date, or language of publication. Data related to *D. rotundus* attacks on humans were excluded.

A total of 52 documents were initially retrieved from Scopus. After applying the inclusion and exclusion criteria and removing duplicates, the final sample consisted of 31 documents from Scopus, 48 documents from other databases, 2 YouTube videos, and 3 camera trap images found online. Although this study did not formally adhere to the PRISMA guidelines (Preferred Reporting Items for Systematic Reviews and Meta-Analyses), the information search followed a systematic, replicable, and explicit approach. Inclusion and exclusion criteria were defined, a structured search strategy was employed, and the document screening process was recorded. For future systematic reviews with clinical or epidemiological purposes, it is recommended to apply the full PRISMA protocol.

### Data Extraction and Analysis

From each document, the following data were extracted: prey species, captivity status (yes/no), lead author, publication date, country of the report, geographic location, associated biome (according to data provided by NatureServe (https://www.natureserve.org/) and the corresponding DOI). Prey observed under captive conditions were recorded separately to distinguish the ecological context.

The analysis of the collected information was conducted using R software (https://www.r-project.org/). In R, the packages ggplot2^(17)^ were used for data visualization, sf^(18)^ for spatial data processing, readxl^(19)^ for importing data from Excel, and rplot for additional graphical presentation of results. The geographic map was created using the geom_point() function from the ggplot2 package, based on the geographic coordinates extracted directly from the predation reports included in the sample. This map allowed the visual representation of *D. rotundus* attack occurrences in relation to the biomes described in each inspected article.

Additionally, to assess whether there was a relationship between the taxonomic class of the prey (mammal or bird) and their status (wild or domestic), a Chi-square (χ^2^) test of independence was applied using the chisq.test() function in R software (v4.2.2). Documents retrieved from Scopus were imported into VOSviewer (https://www.vosviewer.com/) to perform a keyword co-occurrence analysis. This type of analysis has been supported by recent studies, such as Moral-Muñoz et al.,^(20)^ who highlight its usefulness for identifying thematic and collaboration patterns in scientific publications.

## Results

**Figure 1.**
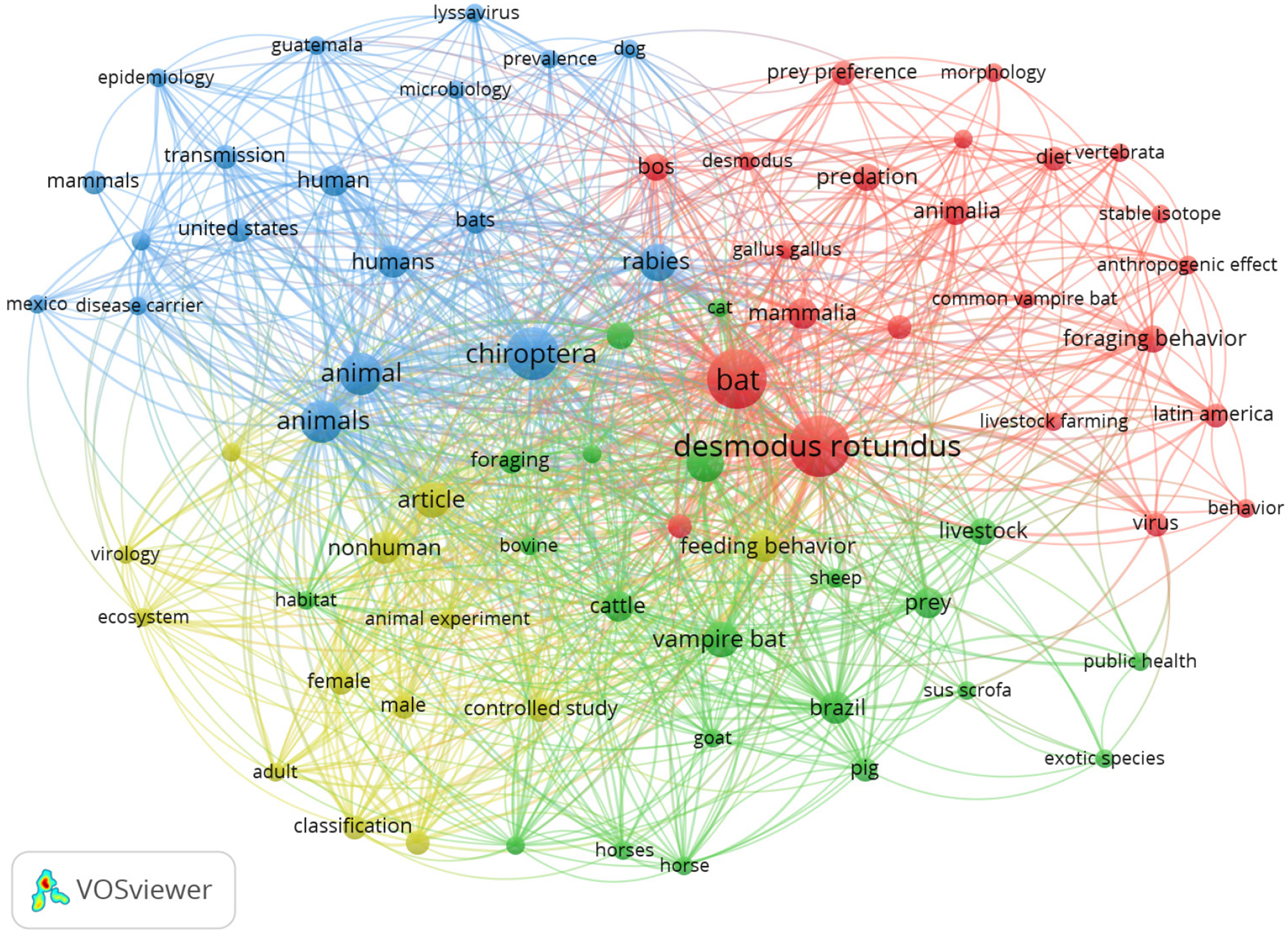
*Keyword co-occurrence networks of the selected articles*. Clusters highlighted in blue (aspects related to the epidemiology of bat-transmitted rabies), red (*D. rotundus* and prey preferences, feeding and hunting behavior), green (the relationship between rabies virus, vampire bats, and their interaction with livestock), and yellow (methodological and taxonomic aspects).

The earliest reference to *D. rotundus* prey dates back to 1940, while the most recent references were from 2023, covering 83 years. During this period, an increasing though, not constant trend was observed in the publication of articles on *D. rotundus* predation, with a notable rise over the past 10 years. Of the 371 keywords collected, only 70 appeared at least twice. The six most frequent keywords were *D. rotundus* (n = 20), bat (n = 19), Chiroptera (n = 15), animal (n = 10), rabies (n = 8), vampire bat (n = 7), and feeding behavior (n = 6) (Figure 1). Clustering of the keywords resulted in four main clusters: research fields focused on (i) bats (red), (ii) human health and epidemiology (blue), (iii) livestock (green), overlapping with (iv) ecology (yellow). Each cluster represents a thematic group defined by the co-occurrence of key terms. (Figure 1).

From the collected documents, a total of 38 prey species were recorded (Table 1), of which 22 (55 %) were wild mammals, ten (25 %) were domestic mammals, six (15 %) were domestic birds, and two (5 %) were wild birds. Among domestic species, most reports involved predation on cattle (n = 21) and pigs (n = 7), while the most frequently cited wild species were tapir (*Tapirus terrestris* n = 5, *Tapirus bairdii* n = 3), wild boar (*Sus scrofa* n = 6), and red brocket deer (*Mazama americana* n = 5) (Table 1). No statistically significant relationship was found between prey type (domestic or wild) and the class (bird or mammal) of animals preyed upon by *D. rotundus* (χ^2^ = 3.444, df = 1, P = 0.063).

For wild and domestic prey reported under captive conditions, 35 records were found (Table 2), including eight reptiles (22.86 %), one amphibian (2.86 %), 13 wild mammals (37.14 %), one domestic mammal (2.86 %), and 12 wild birds (34.29 %).

Several reports do not correspond to direct bites or attacks and should therefore be interpreted with caution (Table 2).

**Figure 2.**
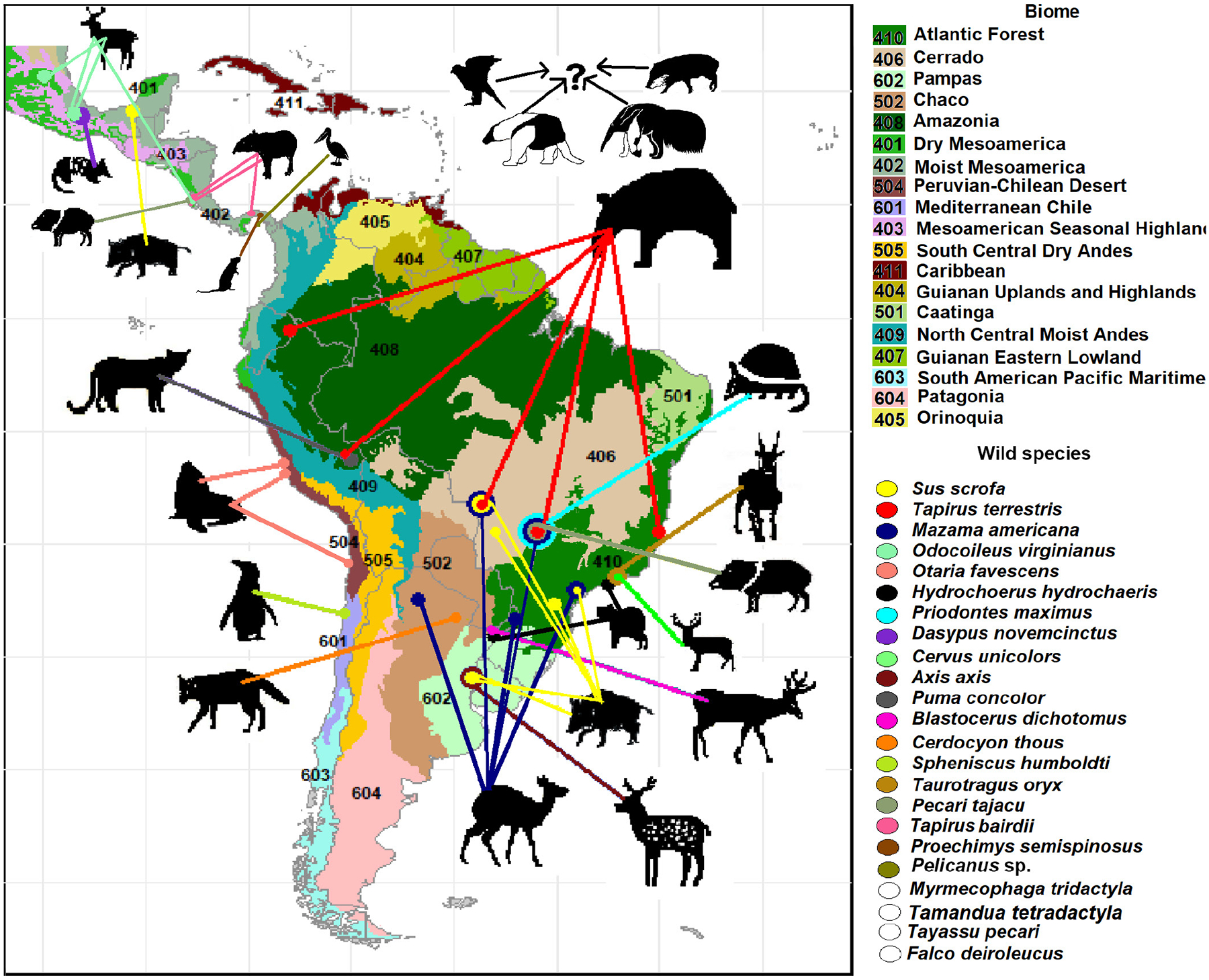
Distribution of *D. rotundus* predation by wild prey species. The map shows the locations where predation by *D. rotundus* was reported, with each prey species represented by a unique color. The map is divided by geographical regions, countries, and biomes (color). Species without precise event locations or cases where camera traps did not allow confirmation of the bat species responsible are also indicated.

In terms of geographical distribution, the biome with the highest number of predation reports on wild prey in the field was the Atlantic Forest (26%), followed by the Mesoamerican Humid Forest (13 %), the Mesoamerican Dry Forest (13 %), Cerrado (10 %), Chaco (8.6 %), pampas (8.6 %), Amazonia (6.5 %), the Chilean–Peruvian Desert (4.3 %), and the Mediterranean Chile (2.1 %) [Figure 2]. The wild species with the widest distribution of reports was the wild boar (*Sus scrofa*), followed by the tapir (*T. terrestris* and *T. bairdii*). For most species, reports of predation by D. rotundus were limited to one or two countries (Figure 2).

## Discussion

The results of this study reveal a remarkable diversity of *D. rotundus* prey species across its geographic range in countries such as Brazil, Argentina, Peru, Chile, Ecuador, Panama, and Mexico. Several species of mammals and birds were identified, including iconic species such as the mountain lion (*Puma concolor*),^(58)^ the giant armadillo (*Priodontes maximus*),^(47)^ and the South American sea lion (*Otaria flavescens*).^(53)^ Greenhall and Schmidt (1988) suggested that it is unlikely for *D. rotundus* to target predatory species as prey, since these animals are aggressive, alert, and nocturnal, making such interactions difficult.^(55)^ However, our data suggest that attacks by *D. rotundus* on predators cannot be entirely ruled out.

The observations by Greenhall and Schmidt were conducted in captivity, which could have influenced the predators’ behavior.^(64)^ An example that challenges this conclusion is the capture, by a camera trap, of a *D. rotundus* bat feeding on a mountain lion (*Puma concolor*).^(58)^ This finding suggests that, in the wild, pumas may indeed be considered prey of *D. rotundus*. Our results are consistent with previous research highlighting the wide range of prey exploited by *D. rotundus*. In their report, Brown and Escobar^(13)^ failed to include three captive prey species: the spiny-tailed iguana (*Ctenosaurus*), the cane toad (*Rhinella marina*), and the ringtail cat (*Bassariscus*). However, the interactions of *D. rotundus* with these species might reflect curiosity rather than predatory attempts or biting behavior.^(55)^ To date, there are no reports, either in captivity or in the wild, providing evidence of *D. rotundus* predation on these species.

Among the potential wild prey of *D. rotundus*, species such as Madidi titi (*Callicebus aureipalatii*),^(24)^ black-capped squirrel monkey (*Saimiri boliviensis*),^(24)^ and South American coati (*Nasua nasua*)^(48)^ should be considered. However, it is uncommon for *D. rotundus* to target predators as food sources—for example, the South American Coati —since these animals are alert, aggressive, and nocturnal. This conclusion is supported by captive observations in which predators such as opossum (*Didelphis* spp.), coyote (*Canis latrans*), raccoon (*Procyon* spp.), South American coati, striped skunk (*Mephitis mephitis*), wildcat (*Leopardus* spp.), and red-shouldered hawk (*Buteo lineatus*) were observed preying upon *D. rotundus* after the vampire bat attempted to feed on them.^(55)^

A case was reported in which a vampire bat preyed upon an orange-breasted falcon (*Falco deiroleucus*). However, the identity of the aggressor species remains inconclusive, as the predation event appears to be more associated with *D. ecaudata* than with *D. rotundus*.^(65)^ This study does not rule out the possibility of such an attack and records the falcon as a potential prey species. In general, the vampire bat tends to prefer animal species larger than itself. For example, in captivity, this behavior was observed when it was offered a white mouse and a vole (*Microtus*), both smaller than *D. rotundus*. Only attempts of predation were observed; rather, the prey was attacked without the bat attempting to feed its blood.^(55)^ Such interactions reinforce the need to distinguish between active predation and defensive encounters, particularly in artificial environments

From an ecological perspective, the wide range of mammals and birds identified as prey demonstrates the adaptability of *D. rotundus* to diverse habitats. It has been documented that *D. rotundus* shows a preference for agricultural landscapes with forested areas and riparian zones.^(66)^ This type of landscape aligns with the findings of this study, which reports predation events in biomes such as the Atlantic Forest and the dry and humid forests of Mesoamerica. These biomes are characterized by warm climates with distinct wet and dry seasons, as well as a rich biodiversity of flora and fauna.^(67)^ Such ecosystems provide an abundant availability of prey for *D. rotundus*. In addition to facilitating access to domestic prey, these environments harbor a diverse array of wild fauna that can serve as alternative food sources and potential epidemiological reservoirs.

The identification of these prey species has significant implications for the management of wildlife rabies. By prioritizing high-risk species and areas for *D. rotundus* attacks, the findings of this study provide valuable guidance for effective surveillance and control strategies aimed at limiting rabies transmission in the regions studied. It is important to consider these predator–prey interactions when implementing vaccination programs and other control measures. According to Brown and Escobar^(13)^ and Voigt and Kelm,^(42)^ domestic species are more frequently affected by *D. rotundus* predation compared to wild species. This preference may be explained by the abundance of domestic animals and their limited defensive capacity, as observed in cattle, horses, goats, and sheep, when faced with predation by *D. rotundus*.^(32, 68)^

Similarly, domestic animals tend to be sedentary, less vigilant, and more closely monitored, making them more susceptible to predation by *D. rotundus* and easier to document.^(66)^ However, the results of this study show that approximately 60 % of reported prey correspond to wild species. This difference may be due to the data collection methodology, as the study relied on previously reported records rather than a field study with a probabilistic design. Therefore, the observed patterns could be influenced by visibility factors or sampling biases. Complementary analyses of prey abundance are suggested to strengthen this conclusion.

Monitoring domestic species such as *Bos taurus* (Bovidae, Linnaeus, 1758), *Canis familiaris* (Canidae, Linnaeus, 1758), and *Felis catus* (Felidae, Schreber, 1775) is crucial for the prevention and control of rabies transmission to humans. However, given the high proportion of records associated with wild prey in this study, it is also essential to strengthen surveillance of feral and wild species such as *Sus scrofa*, *Dasypus novemcinctus* (Dasypodidae, Linnaeus, 1758), and *Odocoileus virginianus* (Cervidae, Zimmermann, 1780), considering their potential roles as pathogen reservoirs and transmitters in rural and natural environments. *Sus scrofa*, *Dasypus novemcinctus*, and *Odocoileus virginianus* are common prey of *D. rotundus*, which increases the risk of pathogen transmission to hunters and hunting dogs.^(50)^

This exposure highlights the need to monitor and assess the presence of rabies and other zoonotic pathogens in game animals. Integrating game species into surveillance programs would enhance understanding of pathogen transmission dynamics and facilitate the implementation of more effective preventive and control strategies. This study is based on report-derived data, which may be subject to biases associated with the detection and reporting of *D. rotundus* attacks. Variability in bat and prey populations across time and space can influence the observed patterns. The implementation of tools such as camera traps,^(46)^ molecular analysis of bat feces,^(22)^ genetic markers^(69)^ and stable isotope analysis^(42)^ are useful for refining prey records.

The analysis of prey and predation behavior provides insights to inform on biodiversity conservation and management strategies. Interventions on landscapes and wild populations have positive effects on reducing rabies. This study serves as a reference for researchers and professionals interested in the ecology and conservation of *D. rotundus* and its impact on rabies ecology. Our results may be influenced by the number of studies conducted in each biome and prey species. This bibliometric report could underestimate the actual frequency and predation preferences in certain regions and prey species.

Furthermore, wild and domestic prey vary according to the biome, which could partly explain differences in predation frequency across different biomes. Predation frequency may also vary depending on the geographical distribution and abundance of different prey species. Finally, it is not always possible to track predator–prey interactions through direct observation^(69)^, so this report is biased toward prey species that are easier to monitor and detect.

## Conclusions

*Desmodus rotundus* has a broad diet, including species of different sizes and trophic positions (herbivores and carnivores). To further understand the ecology of *D. rotundus*, experimental studies are recommended to evaluate the effect of prey on the temporal and epidemiological dynamics of rabies associated with *D. rotundus*. Additionally, it would be beneficial to explore environmental and behavioral factors that may influence the prevalence and attack patterns of these hematophagous bats across different ecosystems. Finally, understanding how the availability of domestic prey affects the expansion of their distribution range could help predict the direction and rate of *D. rotundus* invasion into new areas.

## Figures and Tables

**Table 1.** Predation by *Desmodus rotundu*s on wild and domestic prey

Prey	Class	Sprecies	Common Name	Reference
Domestic	Bird	*Anser cygnoide*,	Swan goose	21
Domestic	Bird	*Numida meleagris*	Helmeted Guineafowl	21
Domestic	Bird	*Cairina moschata*	Muscovy duck	21
Domestic	Bird	*Meleagris gallopavo*	Wild turkey	21
Domestic	Bird	*Pavo cristatus*	Common peafowl	21
Domestic	Bird	*Gallus gallus domesticus*	Poultry fowl	21, 22, 23, 24
Domestic	Mammal	*Bos taurus*	Cattel	21
Domestic	Mammal	*Bubalus bubalis*	Domestic water buffalo	25
Domestic	Mammal	*Equus asinus*	Donkey	22, 23, 24
Domestic	Mammal	*Equus ferus caballus*	Horse	21, 22, 26, 27, 28
Domestic	Mammal	*Capra aegagrus hircus*	Domestic goat	2, 21, 25, 27, 28, 29
Domestic	Mammal	*Sus scrofa domestica*	Domestic pig	21, 22, 23, 24, 26, 27, 30
Domestic	Mammal	*Ovis orientalis aries*	Domestic sheep	2, 27 28
Domestic	Mammal	*Felis catus*	Domestic cat^a^	31, 32
Domestic	Mammal	*Canis lupus familiaris*	Dog	21, 22, 28, 33
Domestic	Mammal	*Bos* spp.^b^	Cattle	2, 16, 21, 22, 23, 24, 25, 26, 28, 30, 34, 35, 36, 37, 38, 39, 40, 41, 42, 43
Wild	Bird	*Pelicanus* sp.^b^	Pelican	44
Wild	Bird	*Spheniscus humboldti*	Humboldt penguin	45
Wild	Mammal	*Dasypus novemcinctus*	Nine-banded armadillo	46
Wild	Mammal	*Priodontes maximus*	Giant Armadillo	47
Wild	Mammal	*Hydrochoerus hydrochaeris*	Capybara	43, 48
Wild	Mammal	*Blastocerus dichotomus*	Marsh deer	43
Wild	Mammal	*Axis axis*	Axis deer	49
Wild	Mammal	*Mazama americana*	Red brocket deer	30, 43, 47
Wild	Mammal	*Taurotragus oryx*	Common eland	21
Wild	Mammal	*Sus scrofa*	Wild pig	30, 49, 50, 51, 52
Wild	Mammal	*Otaria flavescens*	South American sea lion	53, 54
Wild	Mammal	*Artibeus lituratus*	Frugivorous bat^c^	27
Wild	Mammal	*Artibeus jamaicensis*	Jamaican fruit bat^c^	55
Wild	Mammal	*Pecari tajacu*	Collared peccary	47, 56
Wild	Mammal	*Tayassu pecari*	White-lipped peccary	57
Wild	Mammal	*Myrmecophaga tridactyla*	Giant anteater	57
Wild	Mammal	*Puma concolor*	Mountain lion	58
Wild	Mammal	*Proechimys semispinosus*	Spiny rat	59
Wild	Mammal	*Cervus unicolors*	Sambar deer	21
Wild	Mammal	*Tapirus terrestris*	Lowland tapir	23, 35
Wild	Mammal	*Tapirus bairdii*	Baird’s tapir	56, 60, 61, 62
Wild	Mammal	*Tamandua tetradactyla*	Southern Tamandua	58
Wild	Mammal	*Odocoileus virginianus*	White-tailed deer	41, 56, 63
Wild	Mammal	*Cerdocyon thous*	Crab-eating fox	27

a In the case of the domestic cat, rather than being prey to the common vampire bat, it is considered a predator.

b The reported species was not specified.

c It is not confirmed that these species are habitual prey of *D. rotundus*; caution in interpretation is advised.

**Table 2.** Predation observations of *Desmodus rotundus* on wild and domestic prey under captive conditions

Prey	Class	Species	Common Name	Reference
Wild	Amphibian	*Rhinella marina*	Cane toad^a^	55
Wild	Reptile	*Ctenosaurus*	Iguana^a^	55
Wild	Reptile	*Boa constrictor*	Common boa	55
Wild	Reptile	*Elaphe* sp.^e^	Rat snake	55
Wild	Reptile	Leptophis ahaetulla	Parrot snake	55
Wild	Reptile	*Crocodylus* ^e^	Cocodrile	55
Wild	Reptile	*Micrurus* sp.^e^	Coral snake	55
Wild	Reptile	*Pseudemys* sp.^e^	Freshwater turtle	55
Wild	Reptile	*Crotalus durissus*	Tropical rattlesnake	55
Domestic	Mammal	*Cavia porcellus*	Domestic guinea pig	55, 61
Wild	Mammal	*Microtus* sp.^e^	vole^b^	55, 61
Wild	Mammal	*Neotoma* sp.,^e^	Woodrat^b^	55, 61
Wild	Mammal	*Dasypus* sp.^e^	Armadillo	55
Wild	Mammal	*Coendou* sp.^e^	Porcupine	55
Wild	Mammal	*Sylvilagus* sp.^e^	Rabbit	55
Wild	Mammal	*Artibeus jamaicensis*	Jamaican fruit bat	55
Wild	Bird	*Columba livia domestica*	Domestic pigeon	55
Wild	Bird	*Fringillidae* sp.^e^	Finch	55
Wild	Bird	*Dendrocolaptinae* ^e^	Woodcreeper	55
Wild	Bird	*Icteridae* sp.^e^	Blackbird	55
Wild	Bird	*Mimidae* sp.^e^	Mockingbird	55
Wild	Bird	*Picidae* sp.^e^	Woodpeaker	55
Wild	Bird	*Pipridae* sp.^e^	Manakin	55
Wild	Bird	*Psittacidae* sp^e^	Parrot	55
Wild	Bird	*Thraupidae* sp.^e^	Tanger	55
Wild	Bird	*Turdidae* sp.^e^	Thrush	55
Wild	Bird	*Tyrannidae* sp.^e^	Palm tyrant	55
Wild	Mammal	*Didelphis* sp.^e^	Common opossum^c^	55
Wild	Mammal	*Canis* sp.^e^	Coyote^c^	55
Wild	Mammal	*Procyon* sp.^e^	Raccoon^c^	55
Wild	Mammal	*Nasua nasua*	South American coati^c^	55
Wild	Mammal	*Mephitis mephitis*	Striped skunk^c^	55
Wild	Mammal	*Felis silvestris*	Wildcat^c^	55
Wild	Bird	*Buteo lineatus*	Red-shouldered hawk^c^	55
Wild	Mammal	*Bassariscus* ^e^	Ringtail^d^	55

a No bats were observed biting any toads or iguanas. The bats slowly approached the toads, but when the toads jumped, the bats flew away. The bats did not notice the iguana unless it moved.

b No predation attempts were observed; instead, aggression toward this prey was noted, including bites and skin lacerations, without any feeding interaction among them.

c Although they were attacked by D. rotundus, the prey eventually managed to devour the bats.

d No attempts were made to catch the bats, and the bats were also unable to bite the animal.

e Species not specified.

## Data Availability

Relevant data are included within the manuscript and its supporting information files. Additional data are available on Zenodo and can be accessed at the following link: https://zenodo.org/records/13370443

## References

[1] Letko M, Seifert SN, Olival KJ, Plowright RK, Munster VJ. Bat-borne virus diversity, spillover, and emergence. Nature Reviews Microbiology. 2020;18(8):461–471. doi: 10.1038/s41579-020-0394-z. 32528128 PMC7289071

[2] Benavides JA, Velasco-Villa A, Godino LC, Satheshkumar PS, Niño R, Rojas-Paniagua E, Shiva C, Falcón N, Streicker DG. Abortive vampire bat (*sic*.) rabies infections in Peruvian peridomestic livestock. PLoS Neglected Tropical Diseases. 2020;14(6):e0008194. doi: 10.1371/journal.pntd.0008194. 32598388 PMC7351222

[3] Stoner-Duncan B, Streicker DG, Tedeschi CM. Vampire bats and rabies: toward an ecological solution to a public health problem. PLoS Neglected Tropical Diseases. 2014;8(6):e2867. doi: 10.1371/journal.pntd.0002867. 24945360 PMC4063729

[4] Corrêa Scheffer K, Fernandes de Barros R, Iamamoto K, Mori E, Miyuki Asano K, Achkar S, *Diphylla ecaudata* y *Diaemus youngi*, biología y comportamiento. Acta Zoológica Mexicana (NS). 2015;31(3):436–445. doi: 10.21829/azm.2015.3131047.

[5] Greenhall AM. Vampire bat control in the Americas; a review and proposed program for action. Bulletin of the Pan American Health Organization. 1974;8(1):30–36. 4421502

[6] Delpietro H, Russo R. Aspectos económicos y epidemiológicos de la agresión del vampiro y de la rabia paralítica en la Argentina y análisis de las propuestas efectuadas para su control. Revue Scientifique et Technique. 1996;15(3):971–984. doi: 10.20506/rst.15.3.964. 9376648

[7] World Health Organization. WHO expert consultation on rabies: second report. Ginebra, Suiza; 2013. https://apps.who.int/iris/handle/10665/85346 24069724

[8] Streicker DG, Recuenco S, Valderrama W, Gomez Benavides J, Vargas I, Pacheco V, Ecological and anthropogenic drivers of rabies exposure in vampire bats: implications for transmission and control. Proceedings of the Royal Society B: Biological Sciences. 2012;279(1742):3384–3392. doi: 10.1098/rspb.2012.0538. PMC3396893 22696521

[9] Schneider MC, Romijn PC, Uieda W, Tamayo H, Silva DFD, Belotto A, Rabies transmitted by vampire bats to humans: an emerging zoonotic disease in Latin America? Revista Panamericana de Salud Pública. 2009;25(3). doi: 10.1590/s1020-49892009000300010. 19454154

[10] Sih A, Ferrari MCO, Harris D. Evolution and behavioral responses to human-induced rapid environmental change. Evolutionary Applications. 2011;4(2):367–387. doi: 10.1111/j.1752-4571.2010.00166.x. 25567979 PMC3352552

[11] Jones BA, Grace D, Kock R, Alonso S, Rushton J, Said MY, Zoonosis emergence linked to agricultural intensification and environmental change. Proceedings of the National Academy of Sciences. 2013;110(21):8399–8404. doi: 10.1073/pnas.1208059110. PMC3666729 23671097

[12] Oro D, Genovart M, Tavecchia G, Fowler MS, Martínez-Abraín A. Ecological and evolutionary implications of food subsidies from humans. Ecology Letters. 2013;16(12):1501–1514. doi: 10.1111/ele.12187. 24134225

[13] Brown N, Escobar LE. A review of the diet of the common vampire bat (*Desmodus rotundus*) in the context of anthropogenic change. Mammalian Biology. 2023;103:433–453. doi: 10.1007/s42991-023-00358-3. PMC10258787 37363038

[14] Blackwood JC, Streicker DG, Altizer S, Rohani P. Resolving the roles of immunity, pathogenesis, and immigration for rabies persistence in vampire bats. Proceedings of the National Academy of Sciences. 2013;110(51):20837–20842. doi: 10.1073/pnas.1308817110. PMC3870737 24297874

[15] Rocha F, Días RA. The common vampire bat *Desmodus rotundus* (Chiroptera: Phyllostomidae) and the transmission of the rabies virus to livestock: a contact network approach and recommendations for surveillance and control. Preventive Veterinary Medicine. 2020;174:104809. doi: 10.1016/j.prevetmed.2019.104809. 31756671

[16] Orlando SA, Panchana VF, Calderón JL, Muñoz OS, Campos DN, Torres-Lasso PR, Risk factors associated with attacks of hematophagous bats (*Desmodus rotundus*) on cattle in Ecuador. Vector-Borne and Zoonotic Diseases. 2019;19(6):407–413. doi: 10.1089/vbz.2017.2247. 30615584

[17] Wickham H ggplot2: Elegant Graphics for Data Analysis. Springer-Verlag New York, 2016.

[18] Pebesma E Simple Features for R: Standardized Support for Spatial Vector Data. The R Journal. 2018;10(1):439–446.

[19] Wickham H, Bryan J. readxl: Read Excel Files. R package version 1.4.3. https://CRAN.R-project.org/package=readxl

[20] Moral-Muñoz JA, Herrera-Viedma E, Santisteban-Espejo A, Cobo MJ. Software tools for conducting bibliometric analysis in science: an up-to-date review. El Profesional de la Información. 2020;29(1):e290103. doi: 10.3145/epi.2020.ene.03.

[21] Costa L, Esbérard CEL. *Desmodus rotundus* (Mammalia: Chiroptera) on the southern coast of Rio de Janeiro state, Brazil. Brazilian Journal of Biology. 2011;71(3):739–746. doi: 10.1590/s1519-69842011000400020. 21881799

[22] Bobrowiec PED, Lemes MR, Gribel R. Prey preference of the common vampire bat (*Desmodus rotundus*, Chiroptera) using molecular analysis. Journal of Mammalogy. 2015;96(1):54–63. doi: 10.1093/jmammal/gyu002.

[23] Chua P, Caroe C, Crampton-Platt A, Reyes-Avila CS, Jones G, Streicker DG, A two-step metagenomics approach for the identification and mitochondrial DNA contig assembly of vertebrate prey from the blood meals of common vampire bats (*Desmodus rotundus*). Metabarcoding and Metagenomics. 2022;6. doi: 10.3897/mbmg.6.78756.

[24] Streicker DG, Allgeier JE. Foraging choices of vampire bats in diverse landscapes: potential implications for land-use change and disease transmission. Journal of Applied Ecology. 2016;53(4):1280–1288. doi: 10.1111/1365-2664.2690. 27499553 PMC4950014

[25] Goodwin GG, Greenhall AM. Una revisión de los murciélagos de Trinidad y Tobago: descripciones, infección de rabia y ecología. Boletín del AMNH. 1961(122)3.

[26] Pj M Preferential prey selection by *Desmodus rotundus* (E. Geoffroy, 1810, Chiroptera, Phyllostomidae) feeding on domestic herbivores in the municipality of São Pedro-SP. Brazilian Journal of Biology. 2014;74(3):579–584. doi: 10.1590/bjb.2014.0086. 25296205

[27] Delpietro H, Lord RD, Russo RG, Gury-Dhomen F. Observations of sylvatic rabies in northern Argentina during outbreaks of paralytic cattle rabies transmitted by vampire bats (*Desmodus rotundus*). Journal of Wildlife Diseases. 2009;45(4):1169–1173. doi: 10.7589/0090-3558-45.4.1169. 19901391

[28] Delpietro H, Konolsaisen F, Marchevsky N, De Boni Russo G. Domestic cat predation on vampire bats (*Desmodus rotundus*) while foraging on goats, pigs, cows, and human beings. Applied Animal Behaviour Science. 1994;39(2):141–150. doi: 10.1016/0168-1591(94)90134-1.

[29] Moya MI, Pacheco LF, Aguirre LF. Relación de los ataques de *Desmodus rotundus* con el manejo del ganado caprino y algunas características del hábitat en la Prepuna de Bolivia. Mastozoología Neotropical. 2015;22(1):73–84. http://www.scielo.org.ar/scielo.php?script=sci_arttext&pid=S0327-93832015000100008&lng=es

[30] Galetti M, Pedrosa F, Keuroghlian A, Sazima I. Liquid lunch-vampire bats feed on invasive feral pigs and other ungulates. Frontiers in Ecology and the Environment. 2016;14(9):505–506. doi: 10.1002/fee.1431. 32313511 PMC7164104

[31] Jiménez JFC, López RDP, García NV. BAT Reservoirs for rabies virus and epidemiology of rabies in Colombia: a review. CES Medicina Veterinaria y Zootecnia. 2017;12(2):134–150. doi: 10.21615/cesmvz.12.2.5.

[32] Delpietro H, Konolsaisen F, Marchevsky N, de Boni Russo G. Domestic cat predation on vampire bats (*Desmodus rotundus*) while foraging on goats, pigs, cows, and human beings. Applied Animal Behaviour Science. 1994;39(2):141–150. doi: 10.1016/0168-1591(94)90134-1.

[33] Torres FD, Valença C, Filho GV. First record of *Desmodus rotundus* in urban area from the city of Olinda, Pernambuco, northeastern Brazil: a case report. Revista Do Instituto De Medicina Tropical De Sao Paulo. 2005;47(2):107–108. doi: 10.1590/s0036-46652005000200010. 15880224

[34] Fernandes MEB, Costa L, de Andrade FAG, Silva LP. Rabies in humans and non-human in the state of Pará, Brazilian Amazon. The Brazilian Journal of Infectious Diseases. 2013;17(2):251–253. doi: 10.1016/j.bjid.2012.10.015. 23477765 PMC9427357

[35] Gnocchi AP, Srbek-Araujo AC. Common vampire bat (*Desmodus rotundus*) feeding on lowland tapir (*Tapirus terrestris*) in an Atlantic forest remnant in southeastern Brazil. Biota Neotropica, 2017;17(3). doi: 10.1590/1676-0611-bn-2017-0326.

[36] Greenhall AM, Schmidt U, López-Forment W. Attacking behavior of the vampire bat, *Desmodus rotundus*, under field conditions in Mexico. Biotropica. 1971;3(2):136. doi: 10.2307/2989817.

[37] Mendoza-Sáenz VH, Navarrete-Gutiérrez D, Jiménez-Ferrer G, Kraker-Castañeda C, Saldaña-Vázquez RA. Abundance of the common vampire bat and feeding prevalence on cattle along a gradient of landscape disturbance in southeastern Mexico. Mammal research. 2021;66(3):481–495. doi: 10.1007/s13364-021-00572-9.

[38] Romero-Sandoval N, Escobar N, Utzet M, Feijoo-Cid M, Martín M. Sylvatic rabies and the perception of vampire bat activity in communities in the Ecuadorian Amazon. Cadernos de Saude Publica. 2014;30(3):669–674. doi: 10.1590/0102-311x00070413. 24714956

[39] Sánchez-Gómez WS, Salas CIS, Córdova-Aldana DI, Erales-Villamil JA. Vampire bat (*Desmodus rotundus*) Abundancy and frequency of attacks to(*sic*.) cattle in landscapes of Yucatan, Mexico. Research Square. 2021. doi: 10.21203/rs.3.rs-639802/v1. 35258761

[40] Schmidt K, Badger DD. Some social and economic aspects in(*sic*.) controlling vampire bats. Proceedings of the Oklahoma Academy of Science. 1979;59:112–114. https://ojs.library.ok-state.edu/osu/index.php/OAS/article/view/5150

[41] Tello-Mera EL, Mandujano S. Primer registro fotográfico de murciélagos hematófagos Desmodus rotundus (Chiroptera: phyllostomidae) alimentándose de *Odocoileus virginianus* (Artiodactyla: cervidae) en la Reserva de la Biosfera Tehuacán Cuicatlán, México. Mammalogy Notes. 2016;3(1):17–19. doi: 10.47603/manovol3n1.17-1.

[42] Voigt CC, Kelm DH. Host preference of the common vampire bat (*Desmodus rotundus*; chiroptera) assessed by stable isotopes. Journal of Mammalogy. 2006;87(1):1–6. doi: 10.1644/05-mamm-f-276r1.1.

[43] Delpietro H, Lord RD, Russo RG, Gury-Dhomen F. Observations of sylvatic rabies in northern Argentina during outbreaks of paralytic cattle rabies transmitted by vampire bats (*Desmodus rotundus*). Journal of Wildlife Diseases. 2009;45(4):1169–1173. doi: 10.7589/0090-3558-45.4.1169. 19901391

[44] Trapido H Observations on the vampire bat with special reference to longevity in captivity. Journal of Mammalogy. 1946;27(3):217. doi: 10.2307/1375429. 20996664

[45] Luna-Jorquera G, Culik BM. Penguins bled by vampires. Journal of Ornithology. 1995;136(4):471–472. doi: 10.1007/bf01651597.

[46] Ríos-Solís JA, López-Acosta JC. Potential attack of the common vampire bat (*Desmodus rotundus*) on nine-banded armadillo (*Dasypus novemcinctus*) in northern Oaxaca, México. Therya Notes. 2021;2(3):147–150. doi: 10.12933/therya_notes-21-52.

[47] Zortéa M, Silva DAS, Calaça AM. (*sic*.)Susceptibility of targets to the vampire bat Desmodus rotundus are proportional to their abundance in Atlantic forest fragments? Iheringia Serie Zoologia. 2018;108(0). doi: 10.1590/1678-4766e2018037.

[48] Gonçalves F, Magioli M, Bovendorp RS, de Barros Ferraz KMPM, Bulascoschi L, Moreira MZ, Prey choice of introduced species by the common vampire bat (*Desmodus rotundus*) on an Atlantic forest Land-Bridge island. Acta Chiropterologica. 2020;22(1):167. doi: 10.3161/15081109acc2020.22.1.015.

[49] Calfayan LM, Bonnot G, Villafañe IEG. Case reports of common vampire bats *Desmodus rotundus* (Chiroptera: phyllostomidae: desmodontinae) attacking wild exotic mammals in Argentina. Notas sobre Mamíferos Sudamericanos. 2019;01(1):001(sic.) 006. doi: 10.31687/saremnms.19.0.05.

[50] Grotta-Neto F, Peres PHF, Piovezan U, Passos FC, Duarte JMB. Hunting practices of feral pigs (*Sus scrofa*) and predation by vampire bats (*Desmodus rotundus*) as a potential route of rabies in the Brazilian pantanal. Austral Ecology. 2020;46(2):324–328. doi: 10.1111/aec.12971.

[51] Hernández-Pérez EL, Castillo-Vela G, García-Marmolejo G, López MS, Reyna-Hurtado R. Wild pig (*Sus scrofa*) as prey of the common vampire bat (*Desmodus rotundus*). Therya. 2019;10(2):195–199. doi: 10.12933/therya-19-685.

[52] Pereira A, Batista CB, Bender D, Bazílio S. Report on *Desmodus rotundus* (Chiroptera, phyllostomidae) feeding on Sus scrofa (Artiodactyla, Suidae) blood (PDF). Boletim da Sociedade Brasileira de Mastozoologia. 2016;77:151–153. https://sbmz.org/wp-content/uploads/2025/05/BolSBMz77_dez2016.pdf

[53] Catenazzi A, Donnelly MA. Sea lion *Otaria flavescens* as host of the common vampire Bat *Desmodus rotundus*. Marine Ecology Progress Series. 2008;360:285–289. doi: 10.3354/meps07393.

[54] BBC Earth. Vampire bats feeding on sea lions | The Dark: Nature’s Nighttime World [video]. YouTube. 2020(Sep). https://www.youtube.com/watch?v=319G_351G18

[55] Greenhall AM, Schmidt U. Natural History of Vampire Bats. Boca Raton, US: CRC Press. 1988. pp. 208–214.

[56] Guanacaste Wildlife Monitoring. Vampire Bats-Tico Times [video]. YouTube. 2023(Abr). https://www.youtube.com/watch?v=WbhKi63YGsE

[57] Uieda W Hematophagous bats in the Brazilian Amazon: distribution, human aggression and rabies (PDF). 2016. https://illariy.org/wp-content/uploads/2018/08/Uieda-Hematophagous-bats-im-Brazilian-Amazon.pdf

[58] Kays R Candid Creatures: How Camera Traps Reveal the Mysteries of Nature. Baltimore, US: JHU Press. 2016;153–154.

[59] Malaga Alba A Vampire bat as a carrier of rabies. American Journal of Public Health and the Nation’s Health. 1954;44(7):909–918. doi: doi: 10.2105/ajph.44.7.909. PMC1620809 13171471

[60] eMammal. Baird’s Tapir and common vampire Bat (*Desmodus rotundus*). Flickr. s.f. https://www.flickr.com/photos/smithsonianwild/5178681582/in/photostream/

[61] Amit R, Valverde-Zúñiga N. Bucking and charging defense of Baird’s Tapir (*Tapirella bairdii*) from common vampire bats (*Desmodus rotundus*). Therya Notes. 2022;3(3):147–152. doi: 10.12933/therya_notes-22-87.

[62] Castellanos A, Banegas G. Vampire bats bite lowland tapirs in Yasuni National Park, Ecuador. Tapir Conservation. 2005;24,7. doi: 10.5281/zenodo.22648.

[63] Sánchez-Cordero V, Botello F, Magaña-Cota G, Iglesias JG. Vampire bats, *Desmodus rotundus*, feeding on white-tailed deer, *Odocoileus virginianus*. Mammalia. 2010;75(1):91–92. doi: 10.1515/mamm.2010.065.

[64] Mason G, Clubb R, Latham N, Vickery S. Why and how should we use environmental enrichment to tackle stereotypic behaviour? Applied Animal Behaviour Science. 2007;102(3–4):163–188. doi: 10.1016/j.applanim.2006.05.041.65.

[65] Muela A, Curti M, Seminario Y, Hernández A. An incubating orange-breasted falcon (*Falco deiroleucus*) as host for a Vampire Bat. Journal of Raptor Research. 2011;45(3):277–279. doi: 10.3356/JRR-10-38.1.

[66] Avila-Cabadilla LD, Sanchez-Azofeifa GA, Stoner KE, Alvarez-Añorve MY, Quesada M, Portillo-Quintero CA. Local and landscape factors determining (*sic*.) occurrence of Phyllostomid bats in tropical secondary forests. PloS ONE. 2012;7(4):e35228. doi: 10.1371/journal.pone.0035228. 22529994 PMC3329449

[67] Josse C, Navarro G, Comer P, Evans R, Faber-Langendoen D, Fellows M, Ecological systems of Latin America and the Caribbean: a working classification of terrestrial ecological systems in Latin America and the Caribbean (PDF). NatureServe. 2003. https://www.natureserve.org/sites/default/files/lacecologicalsystems.pdf

[68] Flores Crespo R, Said Fernández S, Burns RJ, Clay Mitchell G. Observaciones sobre el comportamiento del vampiro común (*Desmodus rotundus*) al alimentarse en condiciones naturales. Revista Mexicana de Ciencias Pecuarias. 1(27):39. https://cienciaspecuarias.inifap.gob.mx/index.php/Pecuarias/article/view/4142

[69] Sheppard SK, Harwood JD. Advances in molecular ecology: tracking trophic links through predator-prey food-webs. Functional Ecology. 2005;19(5):751–762. doi: 10.1111/j.1365-2435.2005.01041.x.

